# Efficacy and safety of thread embedding acupuncture for facial expression muscles atrophy after peripheral facial paralysis: study protocol for a randomized controlled trial

**DOI:** 10.1186/s13063-021-05696-6

**Published:** 2021-10-30

**Authors:** Binyan Yu, Lihua Xuan, Yutong Jin, Shan Chen, Shan Liu, Yijia Wan

**Affiliations:** 1 Department of Acupuncture and Moxibustion, The First Affiliated Hospital of Zhejiang Chinese Medical University, Hangzhou, China; 2 Clinical Evaluation and Analysis Center, The First Affiliated Hospital of Zhejiang Chinese Medical University, Hangzhou, China

**Keywords:** facial expression muscles atrophy, peripheral facial paralysis, thread-embedding acupuncture, Randomized controlled trial

## Abstract

**Background:**

Facial expression muscles atrophy is one kind of sequelae after peripheral facial paralysis. It causes critical problems in facial appearance of patient as well as social and psychological problems. This study aims to evaluate the efficacy and safety of Thread-embedding acupuncture (TEA) for the management of facial expression muscles atrophy after peripheral facial paralysis.

**Methods:**

This is a patient-assessor blinded, randomized, sham-controlled trial. A total of fifty-six eligible patients will be randomly divided into TEA (n=28) and sham TEA (STEA) (n=28) groups. Both groups will receive TEA or STEA treatment at the frontal muscle and the depressor anguli oris muscle, at one predefined points once a week for eight weeks. Additionally, both groups will receive traditional acupuncture treatment at ten acupoints (GB20, LI4, LR3, GB12, ST7, SI18, LI20, BL2, SJ23, ST4) twice a week for eight weeks as a concurrent treatment. B-mode ultrasonography will be used to assess the changes in facial expression muscle thickness ratio of the affected/healthy side at baseline and at 10 weeks after screening, as the primary outcome. House-Brackmann Grade and lip mobility score will be measured and analyzed at baseline and 4, 8, 10, and 12 weeks after screening, as secondary outcomes.

**Discussion:**

The study will compare TEA with sham TEA to explore the feasibility for TEA in improving facial expression muscles atrophy after peripheral facial paralysis.

**Trial registration:**

Chinese Clinical Trial Registry, ChiCTR1900027170. Registered on 3 November 2019, http://www.chictr.org.cn/edit.aspx?pid=45173&htm=4

**Supplementary Information:**

The online version contains supplementary material available at 10.1186/s13063-021-05696-6.

## Background and rationale

Peripheral facial paralysis (PFP) is caused by peripheral neuronal lesions of the facial nerve, which can be either primary (Bell’s Palsy) or secondary [1]. PFP patients with level 3-5 injury can develop sequelae [2, 3] based on the Sunderland Classification system [4]. The PFP sequelae include synkinesis, contracture, spasm, crocodile tear syndrome, and facial expression muscles atrophy (FEMA) [5]. FEMA is easily ignored by patients and doctors since it occurs in the deep layer below the skin [5, 6]. However, an experienced doctor can diagnose FEMA at about three months after PFP onset [7]. Moreover, FEMA can persist for an extended period or permanently if not properly treated [5]. FEMA symptoms negatively impact the quality of life by causing psychological or social problems [8–10].

Studies have shown that acupuncture can prevent and alleviate muscular atrophy [11, 12], thus improving muscular functions [13, 14]. Thread-embedding acupuncture (TEA) is a novel subtype of acupuncture treatment that includes the insertion and embedding of certain absorbable medical threads, such as catgut, polydioxanone or polyglycolic acid among others, into subcutaneous tissues or muscles at specific points. Absorbable medical thread provides the function of traditional acupuncture for an extended period through the mechanical and chemical stimulations of the thread. It has been widely used for the treatment of musculoskeletal disease [15–17], obesity [18, 19] and cosmetic problems [20], especially in the reduction of facial wrinkles and improvement of skin elasticity in China and other Asian countries [21]. TEA has also been used for the treatment of PFP sequelae [22, 23].

However, the clinical efficacy of TEA for FEMA after PFP is unknown. TEA has been used for FEMA treatment after PFP in our hospital for many years. However, there is no evidence-based clinical research for this treatment. Many studies have assessed the safety and efficacy of TEA on facial connective tissues [15–21]. This study designed a patient-assessor blinded, randomized, sham-controlled trial to explore the efficacy and safety of TEA for the treatment of FEMA after PFP.

## Methods/design

### Objective

The aim of this study is to assess the efficacy and safety of TEA compared to sham TEA in alleviating FEMA after PFP.

### Study design

This is a patient-assessor blinded, randomized, sham-controlled efficacy trial on TEA for alleviating FEMA after PFP. A total of fifty-six eligible participants will be recruited from the inpatient and outpatient departments of the First Affiliated Hospital of Zhejiang Chinese Medical University. They will be assigned randomly into either the TEA or STEA group. Patients in both groups were given TEA (for TEA group), or STEA (for STEA group), and traditional acupuncture treatments (for both groups) for eight weeks. Regular follow-up will be conducted for up to four weeks after the intervention.

**Fig. 1:**
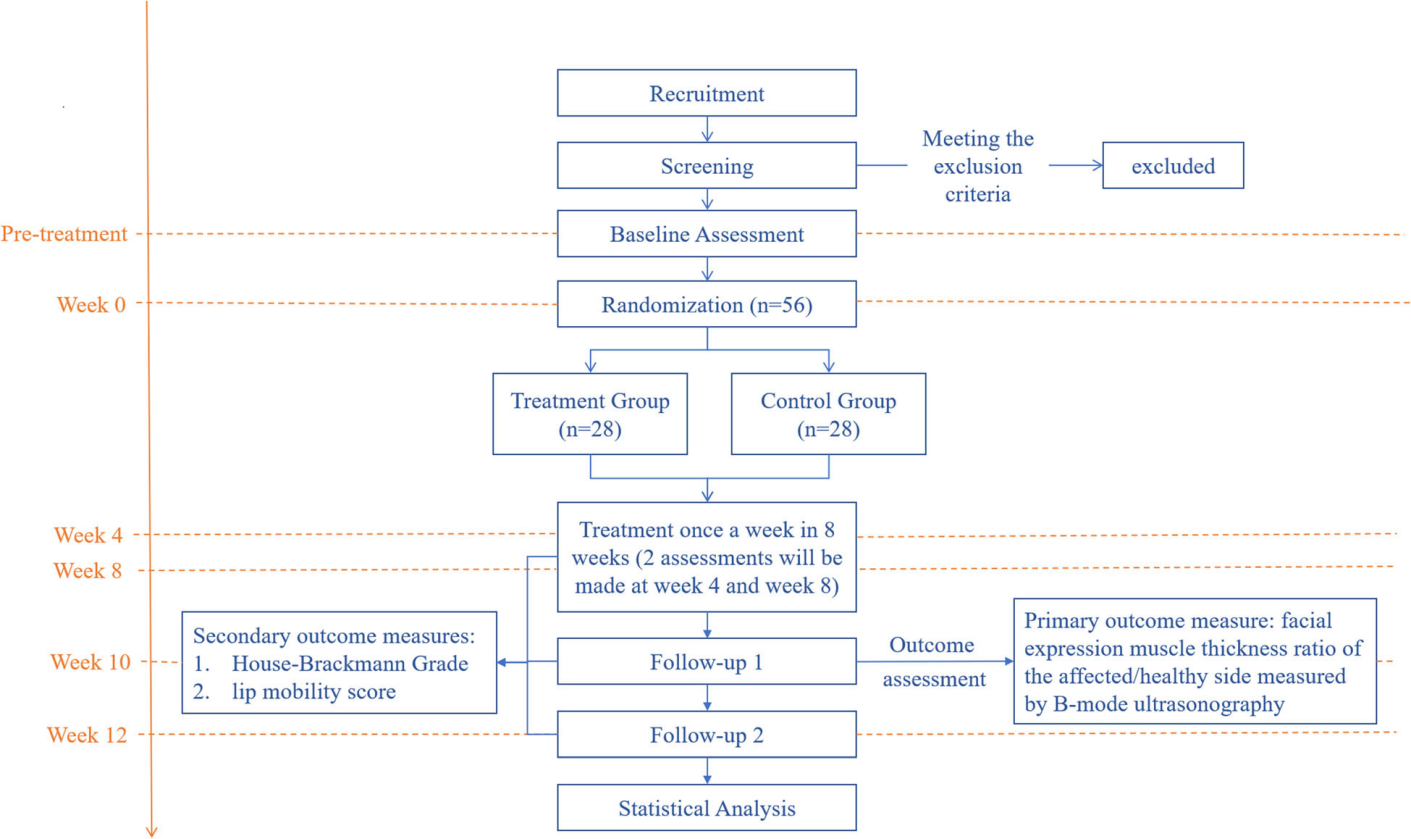
Flow chart for our randomized controlled trial on the efficacy and safety of TEA for FEMA after PFP

**Fig. 2:**
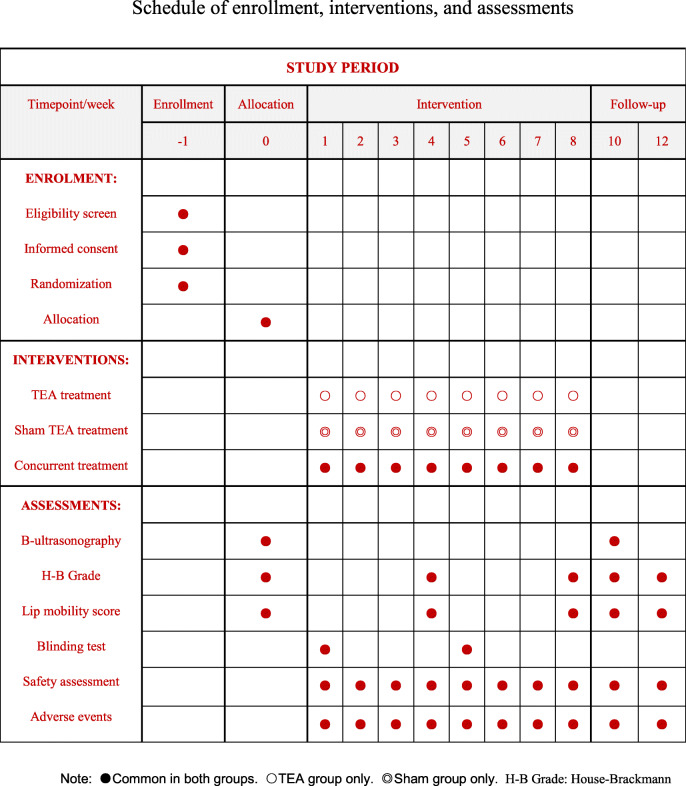
Schedule of enrollment, interventions, and assessments

B-mode ultrasonography will be used to assess the changes in facial expression muscle thickness ratio of the affected/healthy side at baseline and 10 weeks after screening as the primary outcome. House-Brackmann Grade (H-B Grade) [24] and lip mobility score [25] will also be measured and analyzed at baseline and 4, 8, 10, and 12 weeks after screening, as secondary outcomes (Figs. 1, 2). The study protocol was approved by the Ethics Committee of the First Affiliated Hospital of Zhejiang Chinese Medical University on the Use of Human Subjects for Teaching and Research (approval No.2020-K-084–01) and registered in the Chinese Clinical Trial Registry (ChiCTR1900027170). The study design followed the Standard Protocol Items of Recommendations for Interventional Trials (SPIRIT) Checklist (Additional file 1).

### Study subjects

#### Participants and setting

Patients with FEMA after PFP will be recruited. This study will be conducted in the First Affiliated Hospital of Zhejiang Chinese Medical University.

#### Inclusion criteria

Patients will be eligible for the study if they:

1. Male or female patients aged 18–65 years.
2. Patients diagnosed with peripheral facial paralysis ≥3 months before screening.
3. Patients with H-B Grade ≥III.
4. Patients with atrophic facial expression muscles as revealed by B-mode ultrasonography. The muscle thickness of the affected side/healthy side ≤ 90%.

#### Exclusion criteria

1. Patients with central facial paralysis.
2. Patients with bilateral facial nerve palsy or recurrent facial nerve palsy (more than two occurrences).
3. Patients with a history of hypersensitivity to TEA or severe keloid.
4. Patients with contraindications, such as skin diseases and hemostatic disorders (prothrombin time international normalized ratio [PT INR] >2.0 or taking anticoagulant) that inhibit TEA administration.
5. Pregnant or nursing patients.
6. Patients with serious acute or chronic organic diseases and systematic diseases.
7. Patients with mental illness causing the inability to comply with the clinical trial protocol; or any other condition that can render the individual unsuitable for inclusion in the trial, as determined by the principal investigator.

### Elimination criteria

1. Patients included and did not meet the inclusion criteria.
2. Patients who were not excluded and met the exclusion criteria.
3. Eligible participants who are not on any clinical interventions.

### Dropout criteria

1. Poor compliance to the treatment (cannot finish 8-week treatment for personal reasons).
2. Patients experiencing severe adverse events, complications, or special physiological changes.
3. Voluntary dropout.

### Sample size

The sample size was based on previous similar studies [26], and advice from an expert group. The formula below was used to determine the sample size in each group (28 participants) (alpha (significance level), power (1-beta), dropout rate, and group sample ratio were 0.05, 0.90, 10%, and 1:1, respectively). σ indicates standard deviation (23.30) and d represents the mean difference between the two groups (21.54):

$$ \mathrm{n}=2{\sigma}^2{\left({\mathrm{Z}}_{\alpha /2}+{\mathrm{Z}}_{\beta}\right)}^2/{\mathrm{d}}^2 $$

### Recruitment

Subjects will be recruited through either of the following sources: (1) advertisements in newspapers and internet; patients who are interested can contact research staff by phone; or (2) the department of acupuncture and moxibustion of the First Affiliated Hospital of Zhejiang Chinese Medical University receives a high number of patients with FEMA after PFP. Recruitment advertisements will be released through posters and videos displayed on bulletin boards in the outpatient and inpatient lobbies of the First Affiliated Hospital of Zhejiang Chinese Medical University. If they are interested, they can contact research staff by phone. Informed consent will be obtained from eligible patients (Additional file 2).

### Allocation

After completing a baseline evaluation, eligible participants will be randomly assigned to either the intervention or the sham-controlled group in a 1:1 ratio by an independent investigator. Subjects in the intervention group will receive TEA treatment, whereas the subjects in the sham-controlled group will receive the sham treatment. Random numbers will be generated by a statistician from the Clinical Evaluation and Analysis Centre of The First Affiliated Hospital of Zhejiang Chinese Medical University using SPSS for Windows (Version 22.0; SPSS Inc., Armonk, NY, USA). Sealed opaque assignment envelopes were used for allocation concealment. Those envelopes will be stored in a locker and the key kept by the clinical research coordinator (CRC). CRC will offer the envelopes that correspond to the group allocation to the acupuncturist after completion of recruitment.

### Blinding

To achieve participant and evaluator blinding, the clinical research coordinator (CRC) will be the only one allowed to manage the allocation information as well as offer limited information to each researcher in accordance with their roles. The researchers involved in the study will be blinded to the allocation. In both TEA and STEA groups, the participants exhibited the same responses during the procedure. Throughout the whole treatment, the participants’ eyes will be covered with a blindfold to prevent them from observing the procedures. Data managers, statisticians, therapists, and interviewers will be independent and shall not be allowed to communicate allocations or other important information among each other.

The conditions for unblinding are: (1) if serious adverse events occur, and the participant has to be withdrawn from the trial, or (2) the end of the trial.

### Interventions

Specialists in the acupuncture & moxibustion department with at least three years of clinical experience and a license from the Ministry of Health of the People’s Republic of China will administer the treatments strictly following the detailed procedures. Any acupuncture or physiotherapy that can impact the results will be prohibited during the treatment and the follow-up period. Drugs prescribed for the participants four weeks before the trial will be allowed depending on their effect on outcomes. Information regarding medications administered to the participants will be recorded in the case report form (CRF).

### Standard operating procedures

#### Needle requirements

Disposable sterile TEA devices will be used in accordance with national standards within the validity period.

**Fig. 3:**
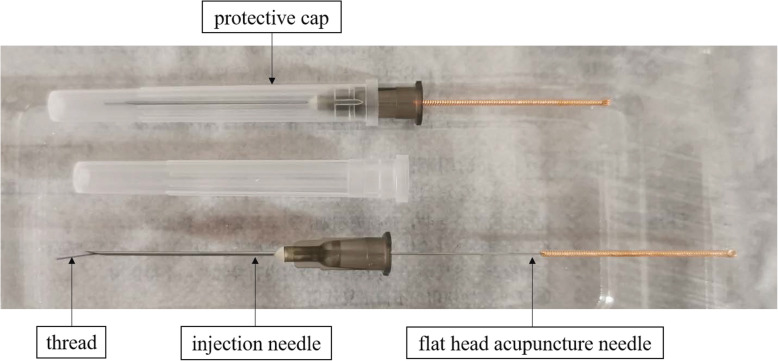
Parts of a thread-embedding acupuncture device

The TEA devices are covered with a protective cap before use and comprised of three parts: 0.7*30TWLB disposable hypodermic needle (Kangbao Medical Equipment co. LTD, Jiangsu, China), 0.4*50mm flat head acupuncture needle (Jiachen Acupuncture Medical Equipment co. LTD, Jiangsu, China) and a 1.0-cm absorbable thread (4-0’, Polyglycolic acid thread made by B. Braun Surgical, S.A, Rubi, Spain) (Fig. 3). The absorbable thread is a major TEA component and is internally buried in the treatment areas. The flat head acupuncture needle is used to push the thread out of the injection needle head into the body tissue.

#### Hygiene of the operator

The operator will be required to sterilize his or her hands using a sanitizer and wear sterile gloves and mask before the TEA and STEA procedures. For the acupuncture procedures, the operator will be required to sterilize his or her hands with a sanitizer before operation.

#### Sterilization of the operation points

The skin on the operation points (5 cm diameter from the acupoint as the center) will be sterilized twice using a cotton swab dipped in 0.45%–0.55% povidone-iodine.

### Procedure

#### TEA group

Participants in this group will receive TEA treatment at two predefined points once a week for eight weeks. Point selection and details of this procedure were based on a consensus of clinical experts and modified from those used in previous study [22].

**Fig. 4:**
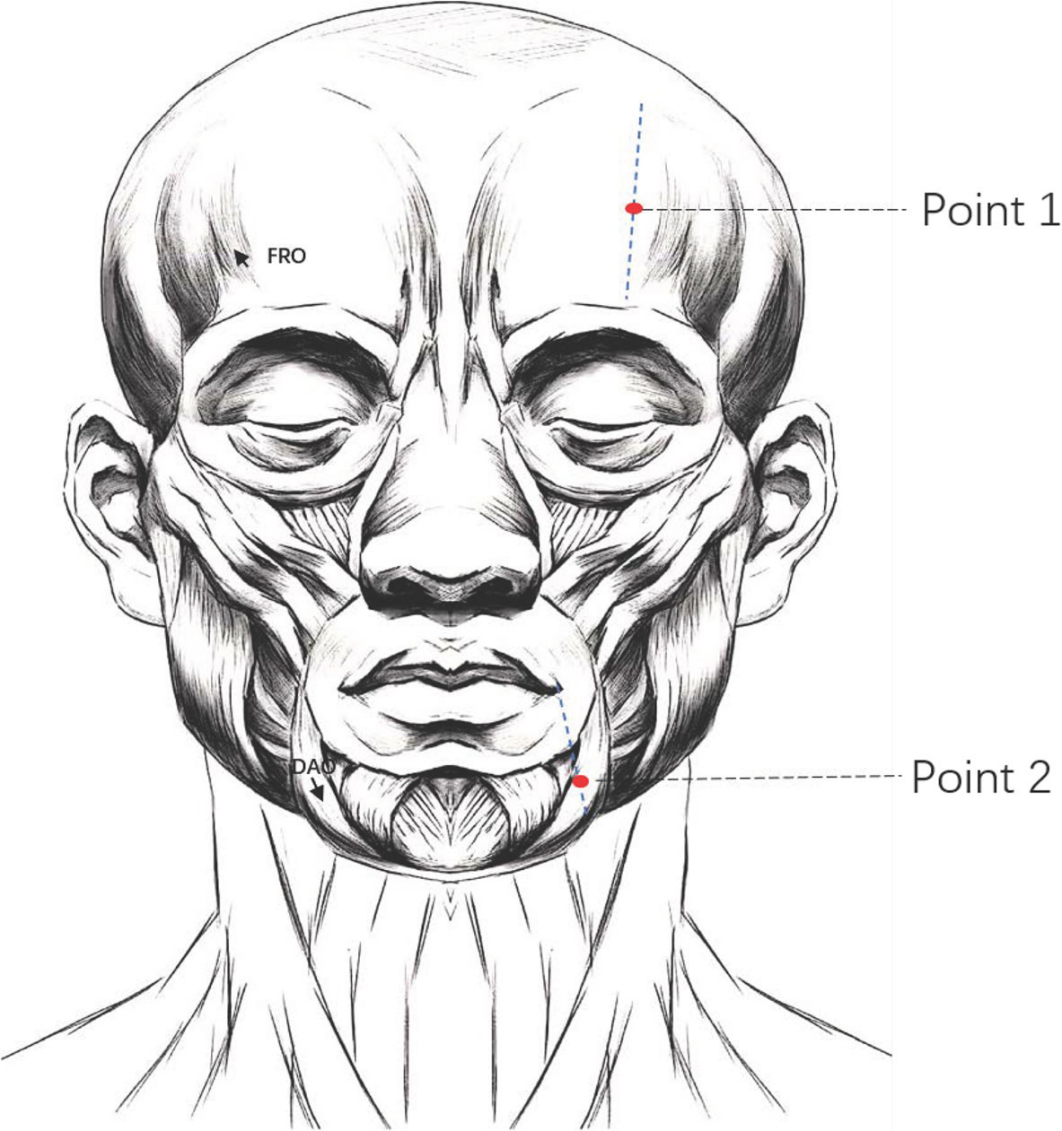
Location of the two operation points

1. Selection of points: Point 1 is located in the frontal (FRO) muscle. Point 1 is in the middle of the line between the eyebrows and the front hairline, forming a straight line from the pupil to the front hairline. Point 2 is located in the depressor anguli oris (DAO) muscle. Point 2 is 1 cm below the oral angle of the line, forming a vertical line to the mandibular margin through the oral angle (Fig. 4).
2. Detailed procedures: After covering the patient’s eyes and sterilizing the skin, practitioners will perform the intervention procedure. The injection needle with thread will be transversely inserted along the line downward from 1 cm above point 1 or 2 in the layer where the two target muscles are. The handle of the flat head acupuncture needle will be pushed to insert the thread in the target muscle after which the injection needle and the flat head acupuncture needle will be removed. Sterile cotton balls will be used to press local pinholes to prevent bleeding.

#### STEA group

Participants in this group will receive STEA treatment at two predefined points (same as TEA group) once a week for eight weeks. Patients in the STEA group will undergo the same procedure as those in the TEA group except that there will be no thread in the TEA device.

Communication will be minimized during interventions to prevent bias except where necessary.

### Concurrent treatment

In China acupuncture has been historically used to treat sequelae after facial paralysis. And research has showed that acupuncture treatment was effective in improving sequelae of facial paralysis [13, 26]. It is highly accepted by most of the patients. So traditional acupuncture will be used as a concurrent treatment for both groups twice a week for eight weeks to improve adherence to the intervention. Moreover, acupuncture treatment will be performed immediately after TEA or STEA treatments. A 0.25*40mm disposable acupuncture needle (Jiachen Acupuncture Medical Equipment co. LTD, Jiangsu, China) will be inserted to a depth of 5 mm and retained for 30min in the following 10 acupoints (Table 1): GB20, LI4, LR3, GB12, ST7, SI18, LI20, BL2, SJ23, ST4.

**Table 1:** Location of the acupoints

Acupoint (standard abbreviation/Chinese nomenclature)	Location
GB20/Fengchi (Affected side)	In the anterior region of the neck, inferior to the occipital bone, in the depression between the origins of sternocleidomastoid and the trapezius muscles
LI4/Hegu (Bilateral Extremity)	on the highest point of the fleshy joining between the index and thumb fingers when they are stretched outwards
LR3/Taichong (Bilateral Extremity)	In the depression anterior to the junction of the first and second metatarsal bones
GB12/Wangu (Affected side)	In the posterior and inferior depression of the mastoid process behind the ear
ST7/Xiaguan (Affected side)	On the face, anterior to the ear, in the depression between the zygomatic arch and the condyloid process of the mandible
SI18/Quanliao (Affected side)	In the lower margin depression of zygomatic bone
LI20/Yingxiang (Affected side)	Next to the ala of the nose
BL2/Cuanzhu (Affected side)	On the medial end of the eyebrow
SJ23/Sizukong (Affected side)	In the depression of the tail of the eyebrow
ST4/Dicang (Affected side)	Outside the corner of the mouth, just below the pupil

### Treatment period

All participants will receive TEA or STEA treatment once a week and concurrent acupuncture treatment twice a week for eight weeks. One of the two acupuncture treatments will be given on the same day and another one will be administered 2-3 days after the first one.

### Criteria for discontinuing interventions

Treatment will be ended if participants meet any of the following conditions:

1. Patient had serious adverse events during or after treatment.

2. Patient was misdiagnosed after randomization.

3. Patient found to be pregnant after randomization.

### Outcome measures

#### Primary outcome measure

As the primary outcome measure, B-mode ultrasonography will be used to measure facial expression muscle thickness ratio of affected/healthy side.

The operator will use the muscle thickness of both sides of frontal muscle and depressor anguli oris muscle to calculate the affected/healthy side ratio.

**Fig. 5:**
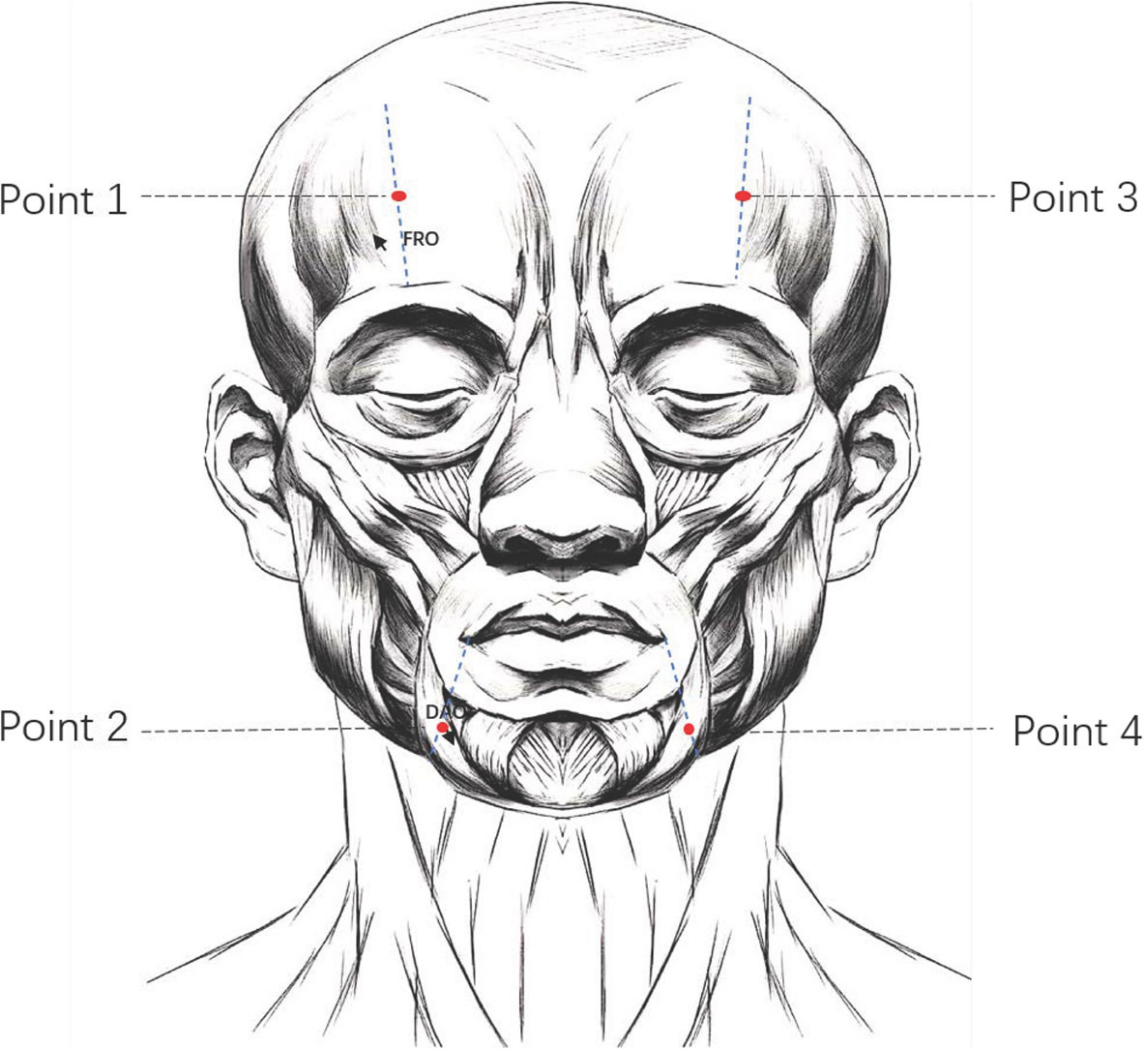
Location of the four operation points

1. Four test points will be on both sides of the frontal muscle and depressor anguli oris muscle (Fig. 5).
2. Detailed procedure: The examination will be performed in a quiet room with low light and at constant room temperature. Participants will lay still for 5 minutes before test. During measurements, participants will lay on the examination bed on their back, keeping their mouths closed and relaxed without making any other expression. An experienced B-mode ultrasonographer will take all measurements.
3. Equipment: GE Logiq9 real-time ultrasonic diagnostic instrument will be used to obtain the measurements. M12L will be used as the probe at a frequency of 5~14MHz and a main frequency of 10MHz.
4. Timepoint of the test: Two measurements will be taken: pre-treatment and at follow-up 1 (week 10).

#### Secondary outcome measures

1. House-Brackmann Grade, the severity of facial paralysis will be assessed using the HB Grade at weeks 0, 4, 8, 10, and 12. Grade I (normal function) to VI (total paralysis) will be evaluated based on the facial function at rest and with effort.
2. Lip mobility score. Lip mobility will be assessed using the lip-length index (LL-index) and snout index (S-index) at weeks 0, 4, 8, 10 and 12. The LL-index will be calculated by determining the percentage change in lip length between grinning and resting. The S-index will be calculated by determining the percentage change in lip length between puckering and resting. Lip length will be measured by determining the inter-commissural length.

### Data collection and management

Two trained research assistants blinded to the treatment groups allocation will record the baseline characteristics of all participants, and the results of the facial expression muscle thickness ratio of affected/healthy side tested by B-ultrasonography at the baseline (week 0) and follow-up 1 (week 10). They will also evaluate and record the HB Grade and Lip mobility score at weeks 0, 4, 8, 10 and 12. Safety assessment and adverse events will be recorded every week starting from week 1 to week 12. Data from this trial will be collected in the CRF. Authorized researchers will record changes in the CRF and provide the date, reason, and signature. Two independent researchers blinded to the group will input the CRF data into an Excel spreadsheet, then cross-check the data.

Hardcopy data will be stored in a secure locker, and electronic data will be encrypted and stored in a specified computer. Only the principal investigator will have access to all documents, protect the electronic documents using a password, and create backups for all documents. The First Affiliated Hospital of Zhejiang Chinese Medical University will be responsible for data storage and management.

### Data processing and statistical analysis

Following the intention-to-treat principle, data processing and analysis will be performed at the Clinical Evaluation and Analysis Centre of The First Affiliated Hospital of Zhejiang Chinese Medical University. These analyses will be performed on the base of a full analysis set (FAS) and a per-protocol set (PPS). All efficacy analyses will be performed using a FAS, which will consist of all patients who passes the randomized stage and who receives at least one intervention. Last observation carried forward (LOCF) analysis will be used for missing data imputation. The PPS will be considered as a supportive analysis. The results of FAS and PPS analyses will be compared to ensure that the results are consistent. A generalized linear mixed-effect model will be used for sensitivity analysis via the PPS. A subgroup analysis will be conducted following the Kellgren–Lawrence grade.

Quantitative results will be described by the mean, standard deviation, median (quartile 3–1, Q3–Q1), minimum, and maximum, while qualitative results will be expressed as total number and percentages. Grade results with rank meaning will be described via both quantitative and qualitative methods.

The chi-square test will be used to compare the primary outcomes between the two groups. The Wilcoxon rank-sum test will be used to compare ranked data and the independent samples t-test for secondary outcome measures to analyze continuous variables. The incidence of adverse events will be determined and compared between the two groups for safety outcomes. A p-value of <0.05 will be considered statistically significant. SPSS Statistics V.22.0 will be used for all analyses. An interim analysis will be conducted when 50% of the randomized patients completed the primary outcome measurement.

### Data monitoring and trial steering committee

A data monitoring committee (DMC) with members from the Clinical Evaluation and Analysis Centre of The First Affiliated Hospital of Zhejiang Chinese Medical University regularly meet to monitor the study data. The DMC will monitor the overall quality and integrity of the data, examine the original CRFs, interview the researchers, verify the record of adverse events, perform an interim analysis, and confirm that the study conforms to the principles of this protocol. A Data and Safety Monitoring Board (DSMB) was also set up to monitor the performance and safety of the trial, which is composed of 5 experts from different fields of the First Affiliated Hospital of Zhejiang Chinese Medical University to ensure the safety of this trial every 6 months. The DMC and DSMB are independent of the trial researchers and have no competing interests. DSMB will reveal a participant’s allocated intervention and make the final decision on whether to terminate the trial. The First Affiliated Hospital of Zhejiang Chinese Medical University will verify participant enrolment, consent, and costs.

### Follow-up

There will be two times of follow-ups after the intervention period. The researchers will contact the participants via phone or WeChat app for successful follow-up.

### Adverse events

Adverse events (AEs) for acupuncture refers to the occurrence of symptoms or diseases that inhibit the purpose of treatment during or after acupuncture treatment. All AEs associated with TEA or acupuncture will be reported to the researcher by the participants or observed by the researcher. The description of AEs will include, time of occurrence, location of the reaction, level of severity, corresponding management and the necessity for patient withdrawal from the trial. AEs of TEA or acupuncture will be classified as local or systemic reactions according to their location.

### Local reactions

Minor bleeding at the needle point.

Subcutaneous bruise.

Subcutaneous haematomas.

Pain in the operated area after operation.

Local allergy in the punctured region after treatment.

Local infection.

Local nodules.

### Systemic reactions

Dizziness or fainting during or after treatment.

Systemic allergy.

Systemic infection.

For patients with common adverse reactions, appropriate medical care will be given to ease local bleeding, bruising and so on.

Severe AEs are defined as symptoms or diseases that result in hospitalization or prolonged hospitalization, disability, a life-threatening situation or even death. In this trial, systemic infection and allergy are two major severe AEs. Severe AEs will be reported to the Ethics Committee within 24 hours. The Ethics Committee will provide medical suggestions for the research team and decide on whether the patient should continue with the ongoing treatment. Proper compensation will be given to cover their medical costs.

### Modification of the protocol

Any modifications to the protocol, including changes to the study objectives, study design, patient population, sample sizes, study procedures or significant administrative aspects, will require a formal application to the Zhejiang Provincial Administration of Traditional Chinese Medicine as well as the Chinese clinical trial registry.

### Dissemination

Initial data will be accessible through the Research Manager (ResMan). The results of this study will be published in open-access and peer-reviewed journals or presented at relevant conferences.

## Discussion

Acupuncture is widely used to treat PFP after the acute stage in China [27–29]. It is commonly used to treat FEMA after PFP. However, many patients find it difficult to adhere to it since it requires about three treatments per week. TEA is a new type of acupuncture with a low treatment frequency (once a week). The suture threads, such as polyglycolic acid which has good biodegradability and biocompatibility [30], are widely used in TEA in China. The TEA-embedded thread can increase the tensile strength and stimulate myoblast formation in connective tissues [21, 31], which is considered to be the basis for the clinical application of this treatment.

Herein, STEA is used to determine the effect of specific intervention factors. STEA will be applied to minimize the needle insertion effect since the penetration of traditional acupuncture can be used to treat muscular atrophy. Participants, assessors, and statisticians will be blinded to the groups to minimize comparison bias.

B-ultrasonography is widely used to measure muscle or other soft tissues and disease diagnosis [32]. It is a sensitive detection method for facial muscle thickness and safe, non-invasive, simple to operate, and cost-effective [33–35]. Studies have shown that normal humans have no significant differences in bilateral expression muscle thickness [36, 37]. Therefore, the thickness of healthy facial expression muscles can be used to detect the atrophic facial expression muscle side. Herein, the test was performed at weeks 0 and 10. The final test will be set at two weeks after treatment because improvements of the muscle structure and strength need a long time for recovery.

Moreover, facial expression muscle function recovery is essential for the evaluation of FEMA treatment using TEA. The physical functions will be assessed via H-B Grade and lip mobility score as the secondary outcome measures.

However, this study has some limitations since it is not designed for a stratified study. There are differences in FEMA recovery after PFP with different disease courses. Stratified observation can screen out the best efficacy group with certain disease courses, giving accurate guidance of TEA timing for clinical treatment of FEMA after PFP.

In summary, this study will evaluate the efficacy and safety of TEA for FEMA after PFP. These findings will enhance understanding of TEA as a therapeutic option for FEMA patients after PFP.

### Trial status

This study protocol version number is 1.0, dated 10 July 2019. The participants have been recruited for the present study since October 2020. Ten patients are under treatment. And the recruitment will be completed in March 2022.

## Supplementary Information

**Additional file 1.**

**Additional file 2.**

## Data Availability

The datasets used and analyzed during the current study are available from the corresponding author on reasonable request.

## Abbreviations

PFP: Peripheral facial paralysis
FEMA: facial expression muscles atrophy
TEA: thread-embedding acupuncture
STEA: sham thread-embedding acupuncture
CRF: Case report form
H-B Grade: House-Brackmann Grade
LL-index: lip-length index; S-index: snout index
CRC: clinical research coordinator
FAS: full analysis set
PPS: per-protocol set
LOCF: last observation carried forward
DMC: data monitoring committee
DSMB: Data and Safety Monitoring Board
AEs: adverse events
