# Supplementary material for: Efficacy and safety of thread embedding acupuncture for facial expression muscles atrophy after peripheral facial paralysis: study protocol for a randomized controlled trial
Source: Trials. 2021 Oct 30;22:755. doi: 10.1186/s13063-021-05696-6 (PMC8559375; doi:10.1186/s13063-021-05696-6)
Supplement: Supplementary file 2 — Additional file 2. [file 13063_2021_5696_MOESM2_ESM.doc]

埋线治疗周围性面瘫后面肌萎缩技术方案的研究

知情同意书·知情告知页

**版本号：2019003**

**版本日期: 2020年9月15日**

亲爱的患者：

您的医生已经确诊您患有周围性面瘫。

我们将邀请您参加一项《埋线治疗周围性面瘫后面肌萎缩技术的研究》的试验性研究，以观察他们对于周围性面瘫后面肌萎缩的疗效和安全性。治疗通过面部萎缩表情肌埋线。

在您决定是否参加这项研究之前，请尽可能仔细阅读以下内容，它可以帮助您了解该项研究以及为何要进行这项研究，研究的程序和期限，参加研究后可能给您带来的益处、风险和不适。如果您愿意，您也可以和您的亲属、朋友一起讨论，或者请您的医生给予解释，帮助您做出决定。

研究介绍

**一、研究背景和研究目的**

周围性面瘫是由面神经核及其以下段面神经损伤导致的面部表情肌功能障碍的疾病。周围性面瘫中发病率最高的贝尔面瘫虽然有一部分患者有自愈倾向，但众多报道显示对于早期未正规治疗，平均年龄较大的患者（大于60岁），只有30%能恢复正常^1^；肌电图检查提示最小振幅占正常振幅的百分比，比值＜10%的患者中仅有13%面神经功能恢复正常^1^；而因肿瘤术后、外伤等原因所致的周围性面瘫，其后遗症比例则远远高于此^2^。在面瘫后遗症中，除了人们熟知的联动、痉挛、鳄鱼泪外，面肌萎缩也是对患者最终容貌有严重影响的后遗症之一^3,4^。由于面肌萎缩发生在皮肤的深面，在面瘫早期很容易被患者和医师忽视，一旦出现肉眼可辨的萎缩，往往已发病3个月以上。患者会诉患侧脸比健侧缩小，此时的面瘫治疗会变得更加复杂和困难。后遗症严重影响患者的生理和心理健康^5^，大大降低患者的生存质量^3,6^。一直以来，人们对面神经疾病的关注热点集中在神经损伤后的早期治疗中，而对于后遗症中的面肌萎缩的研究相对较少。

周围神经损伤后，神经的传导功能障及周边的微循环障碍是失神经肌萎缩的可能机制。改善神经传导功能，提高骨骼肌供氧，适度增加骨骼肌刺激和被动收缩、改善血运是能够延缓肌萎缩的方法。针刺能促进周围神经损伤后传导功能的恢复，改善失神经肌肉微循环障碍，是治疗失神经肌萎缩的有效手段。^7-11^

穴位埋线疗法是针灸治疗方式的创新和发展，该方式应用一次性埋线针在穴位内植入可降解线体，通过线体长期刺激穴位发挥类似传统针灸“留针”的治疗效果，其治疗效应强而持续，一般每1~2周治疗1次，患者接受度高。新型的埋线材料聚羟基乙酸（polyglycolic acid，PGLA）因其生物降解性优异、组织相容性好及不良反应少等特点，长期应用于各类外科手术中，安全性佳^12^。它一方面可以达到持续刺激穴位的目的，另一方面又可以减少免疫反应的发生，因此具有广阔的应用前景。

近年来埋线疗法已广泛应用于各类肌萎缩的治疗中，临床疗效佳，但尚未有关于埋线治疗周围性面瘫后面肌萎缩的报道。^13,14^在长期临床应用实践中我们观察发现，埋线治疗能有效改善周围性面瘫后期面肌萎缩的情况，相较于针刺治疗，其单次治疗时间短，作用时间持久，受到了患者的普遍欢迎。埋线疗法在我国针灸领域应用近30余年，目前已形成了非常规范的诊疗操作技术流程，临床推广的基础好。

我院针灸科长期致力于面神经相关疾病诊治的研究，在临床治疗周围性面瘫方面疗效显著，深受患者欢迎。承担了相关浙江省自然科学基金项目和国家、省部及厅局级项目，完成相关课题6项，成果获得浙江省科学技术二等奖，获国家发明专利1项，发表了9篇相关的研究论文，举办了3期国家级继续教育学习班“面神经疾病诊治进展学习班”，完成了《浙江省中医（中西医结合）单病种诊疗方案》中“面神经炎”的编写，前期工作基础好。

该研究目的：评价埋线治疗周围性面瘫后面肌萎缩技术的有效性及安全性。规范该治疗方法，为该技术的应用和推广提供临床依据，建立疗效评价标准，形成标准操作规程（SOP）和技术规范文本。

伦理委员会已经审议此项研究是遵从赫尔辛基宣言原则，符合医疗道德的。

**二、哪些人不宜参加研究**

(1)中枢性面瘫患者；合并肺心病、呼吸衰竭的患者；

(2)妊娠和哺乳期妇女；

(3)合并严重心脑血管疾病、肝、肾、肺、血液系统疾病和精神疾病；

(4)正在参加其它临床研究者。

**三、如果参加研究将需要做什么**

1. 在您入选研究前，您将接受以下检查以确定您是否可以参加研究：

医生将询问、记录您的病史，对您进行体格检查。

您需要做面部表情肌B超测定、House-Brackmann 面神经功能分级评分、唇部活动度评分等检查。

2. 若您已完成以上检查，将按以下步骤进行研究（按随访时点详细陈述治疗及各检查项目）

研究开始将根据计算机提供的随机数字，决定您接受试验组或对照组方案。参加这项研究的患者分别有50%的可能性被分入这两个不同的组别。您和您的医生都无法事先知道和选择任何一种干预措施。治疗观察将持续8周，继续随访至12周。

①相关症状和体征：治疗前及治疗后第4、8、10及12周记录。

②面部表情肌B超测定：治疗前及治疗后第10周检测。

③其他：治疗后安全性评价和不良事件记录在治疗后至随访期每周均记录一次。盲法检测，在治疗后第1、4周进行。

3. 需要您配合的其他事项

您需要按医生和您约定的随访时间来医院就诊。您的随访非常重要，因为医生将判断您接受的研究措施是否真正起作用。

您需要按医生指导治疗。

在研究期间您不能接受其他周围性面瘫后面肌萎缩的药物及治疗方法。如您需要进行其它治疗，请事先与您的医生取得联系。

**四、参加研究可能的受益**

您和社会将可能从本项研究中受益。此种受益包括您的病情有可能获得改善，以及本项研究可能帮助开发出一种新治疗方法，以用于患有相似病情的其他病人。

您将在研究期间获得良好的医疗服务，享受优先挂号、免费咨询、获得免费针对您的病情的针灸及埋线治疗。

**五、参加研究可能的不良反应、风险和不适、不方便**

埋线治疗的可能副作用：部分患者可能出现晕针、血肿、感染、局部出现肿块等不良反应。

如果您在治疗过程中出现了以上几种情况，我们将会针对您的症状按照针灸中出现这些现象的标准化处理流程对您的情况加以治疗。

尽管到目前为止没有发现该研究方法有其他不良反应，如果在研究中您出现任何不适，或病情发生新的变化，或任何意外情况，不管是否与治疗有关，均应及时通知您的医生，他/她将对此作出判断和医疗处理。

医生和课题组将尽全力预防和治疗由于本研究可能带来的伤害。如果在临床研究中出现不良事件，科研项目所属政府管理部门及医院伦理委员会将会鉴定其是否与本研究有关。研究者/课题组将对与研究相关的损害提供治疗的费用及相应的经济补偿。

您在研究期间需要按时到医院随访，做一些检查，这些都可能给您造成麻烦或带来不方便。

此外，（研究干预）可能出现无效的情况，以及因治疗无效或者因合并其他疾病等原因而导致病情继续发展。在研究期间，如果医生发现本项研究所采取的（研究干预）措施无效，将会中止研究，改用其他可能有效的治疗措施。

**六、有关费用**

如果发生与研究相关的损害，研究者/课题组将支付您的医疗费用。如果您同时合并其他疾病所需的治疗和检查，将不在免费的范围之内。

**七、个人信息是保密的吗？**

您的医疗记录（研究病历/CRF、化验单等）将完整地保存在医院，医生会将化验检查结果记录在您的门诊病历上。研究者或课题组成员、伦理委员会和课题所属政府部门将被允许查阅您的医疗记录。任何有关本项研究结果的公开报告将不会披露您的个人身份。我们将在法律允许的范围内，尽一切努力保护您个人医疗资料的隐私。

除本研究以外，有可能在今后的其他研究中会再次利用您的医疗记录。您现在也可以声明拒绝除本研究外的其他研究利用您的医疗记录。

**八、怎样获得更多的信息？**

您可以在任何时间提出有关本项研究的任何问题。您的医生将给您留下他/她的电话号码以便能回答您的问题。

如果您对参加研究有任何抱怨，请联系医院伦理委员会办公室。

如果在研究过程中有任何重要的新信息，可能影响您继续参加研究的意愿时，您的医生将会及时通知您。

**九、可以自愿选择参加研究和中途退出研究**

是否参加研究完全取决于您的自愿。您可以拒绝参加此项研究，或在研究过程中的任何时间退出本研究，这都不会影响您和医生间的关系，都不会影响对您的医疗或有其他方面利益的损失。

您的医生或研究者出于对您的最大利益考虑，可能会随时中止您参加本项研究。

您可以不参加本项研究，或中途选择退出研究。

如果您因为任何原因从研究中退出，您可能被询问有关您治疗的情况。如果医生认为需要，您也可能被要求进行实验室检查和体格检查。这对保护您的健康十分有利。

**十、现在该做什么？**

是否参加本项研究由您自己决定。您可以和您的家人或者朋友讨论后再做出决定。

在您做出参加研究的决定前，请尽可能向您的医生询问有关问题，直至您对本项研究完全理解。

感谢您阅读以上材料。

如果您决定参加本项研究，请告诉您的医生或研究助理，他/她会为您安排一切有关研究的事务。

请您保留这份资料。

知情同意书·同意签字页

临床研究项目名称：埋线治疗周围性面瘫后面肌萎缩技术的研究

临床研究开展单位：浙江中医药大学附属第一医院

伦理审查批件号： 伦理审查批件右上角

同意声明

我已经阅读了上述有关本研究的介绍，而且有机会就此项研究与医生讨论并提出问题。我提出的所有问题都得到了满意的答复。

我知道参加本研究可能产生的风险和受益。我知晓参加研究是自愿的，我确认已有充足时间对此进行考虑，而且明白：

Ⅰ我可以随时向医生咨询更多的信息。

Ⅱ我可以随时退出本研究，而不会受到歧视或报复，医疗待遇与权益不会受到影响。

我同样清楚，如果我中途退出研究，特别是由于埋线治疗过程的原因使我退出研究时，我会将病情变化告诉医生，完成相应的体格检查和理化检查，这将对我本人和整个研究十分有利。

如果因病情变化我需要采取任何其他的药物治疗，我会在事先征求医生的意见，或在事后如实告诉医生。

我同意伦理委员会或申办者代表及研究质量监察人员查阅我的研究资料。

我同意□ 或拒绝□ 除本研究以外的其他研究利用我的医疗记录和检查结果。

我将获得一份经过签名并注明日期的知情同意书副本。

最后，我决定同意参加本项研究。

受试者签名： 　＿ ＿ ＿ ＿ 年 ＿ ＿ 月 ＿ ＿ 日

受试者联系电话： 手机号：

法定代理人签名（如有）： 日期： 年 月 日

我确认已向患者解释了本研究的详细情况，包括其权利以及可能的受益和风险，并给其一份签署过的知情同意书副本。

研究者签名： 日期：＿ ＿ ＿ ＿ 年 ＿ ＿ 月 ＿ ＿ 日

研究者工作电话： 手机号码：

**浙江中医药大学附属第一医院伦理委员会办公室联系电话：**

0571-87013311
